# Rapid Dilatation of the Female Urethra for the Cure of Cystitis

**Published:** 1884-10

**Authors:** A. R. Davidson

**Affiliations:** Professor Medical Chemistry, Toxicology, Etc., Niagara University. Physician to the Buffalo Hospital of the Sisters of Charity


### Clinical Reports.

### Rapid Dilatation of the Female Urethra for the Cure of Cystitis.

Reported by A. R. Davidson, M. D.

Professor Medical Chemistry, Toxicology, etc., Niagara University. Physician to the Buffalo Hospital of the Sisters of Charity.

Mrs. B., aged 60, mother of twelve children, had enjoyed excellent health up to eighteen months ago, when she commenced to experience pain in the bladder and frequent painful micturition. The irritability of the organ gradually increased, and for the last six months she has been tormented by the imperative necessity of making water every fifteen minutes to one-half hour, enjoying no rest day or night. She had been in the hands of several physicians, and had received the usual treatment for chronic cystitis without obtaining relief, and at last consulted an eminent practitioner of this city, who kindly referred the case to me.

The urine at this time was found to be highly ammoniacal and to contain much blood and pus intermingled with numerous crystals of triple phosphates. The microscope showed, also, soft flocculent masses made up of spindle-shaped cells with very distinct nuclei. Every day the patient passed, per urethra, considerable masses of triple phosphates, very rough and having many branched projections. The quantity of pus, blood and debris from the bladder made it impossible to distinguish kidney epithelium or casts with certainty. The considerable albumen present might be accounted for by the quantity of pus and blood. At the first examination, the extreme sensitiveness of the bladder made a careful sounding impossible, but it was easy to find many small calculi, such as she was daily voiding. A decided thickening and rigidity of the base of the bladder could be felt by vaginal examination, but she complained of so great pain upon any pressure that I deferred a more careful examination until she was etherized.

The patient willingly consented to any operation which would promise a relief from her torment, and on the 13th of August, having placed her under the influence of ether, the sound and vaginal examination of the walls of the bladder quickly demonstrated the absence of any large stone. I therefore proceeded to dilate the urethra, using the little finger first, and following it with the index finger. The base of the bladder was then found to be coated with a phosphatic concretion deposited upon small, reddish, flesh-like masses, which were easily scraped off with the finger nail. The bleeding was not great; as the growth seemed to be confined to the mucous membrane; I have little doubt that it was an innocent villous growth. As Ultzman points out, these do not give rise to a thickening of the coats of the bladder, that is, there is no infiltration of the tissues. Villous cancers, on the other hand, give rise to tumors of the bladder or to thickening, which may be felt through the rectum or the abdominal walls. The patient made a rapid recovery. She complained of some soreness during micturition, and blood and pus were present in the urine for a few days, but the severe pain and frequency of micturition were absent from the moment of the operation. Subsequent treatment consisted of milk diet, regulation of the bowels, iron internally, and, for a week, daily washing out of the bladder. At that time all blood and pus had ceased; the urine was clear and normal in its reaction, and up to the present time (six weeks after the operation) she is free from all untoward symptoms.

The treatment of chronic cystitis by drugs, and its local treatment by injections, are almost always tedious, often unsatisfactory, and not rarely altogether unsuccessful. The treatment here adopted, viz., the rapid dilatation of the urethra, has been condemned by many excellent authorities, and as highly commended by others. It is by no means new, having been recommended and described by Marianus Sanctus in the sixteenth century. Douglass and Bertrandini, in 1769, performed the operation gradually, by tents made from the roots of plants or by sponge covered by parchment, but these early operations were for the extraction of calculi and foreign bodies. Sir Astley Cooper devised a metallic expanding dilator, but modern impetus to the employment of the operation was chiefly given by the late Prof. Simon, of Heidelberg. He recommended the dilatation of the female urethra for the following objects:

1. The diagnosis of diseases of the mucous membrane of the bladder, and of calculus and other foreign bodies.

2. The removal of calculi and foreign bodies.

3. For applying various remedies to the internal surface of the bladder and for treating fissures of the urethra.

4. For the diagnosis of the position and attachment of tumors in the vesico-vaginal septum, and for the removal of tumors, especially papillary growths, from the walls of the bladder.

5. For the discovery and removal of calculi from the vesical extremity of the ureters.

6. For the opening of hæmato-metra, the evacuation of which, between the bladder and rectum, is impossible or dangerous, as, for example, in the case of congenital absence, either partial or complete, of the vagina.

7. For the treatment of vesico-intestinal fistula.

According to Dr. Simon, in adult females the urethra may be safely dilated by means of plugs, having a diameter not exceeding two centimeters. In two cases he carried the dilatation one centimeter further (about one and one-half inch) with the result of having incontinence of urine, though not permanent, in both cases.

The principal opponent to the operation, at the present time, is one whose opinion is entitled to the utmost respect, from his vast gynecological experience. Dr. Emmet, of New York, reports that in eleven cases operated on by himself, two had permanent incontinence and that he had seen this misfortune to follow the operation in at least half a dozen cases in the hands of other surgeons. Moreover, he knew of no benefit from the proceeding in chronic cystitis. On the other hand, Noeggerath, Munde, Goodell, Cronyn of this city, and others, strongly advocate and frequently practice it. It has been suggested as an explanation of the ill results of the operation in Emmet's hands that his index finger has a considerable greater diameter than two centimeters.

The experience of many operators would seem to show that the operation is free from danger of subsequent permanent incontinence of urine, if the urethral tissues are fairly healthy, and the dilatations be not carried to an extent greater than is necessary for the introduction of an index finger of medium size. The ability to thus examine the entire internal surface of the bladder with the finger (by perineal section of the urethra, the male bladder may be almost as easily examined as the female) offers a valuable addition to the means ordinarily employed for the more difficult and otherwise intractable cases of urinary diseases which come before us.

